# The Characteristics of Absorbency Under Load (AUL) for Superabsorbent and Soil Mixtures

**DOI:** 10.1038/s41598-019-54744-4

**Published:** 2019-12-02

**Authors:** Jakub Misiewicz, Krzysztof Lejcuś, Jolanta Dąbrowska, Daria Marczak

**Affiliations:** 1 Wrocław University of Environmental and Life Sciences, Institute of Environmental Engineering, Wrocław, 50-363 Poland; 2 Wrocław University of Life and Environmental Sciences, Institute of Building Engineering, Wrocław, 50-363 Poland

**Keywords:** Environmental impact, Environmental chemistry, Environmental sciences

## Abstract

Various applications of superabsorbent polymers (SAP) include the use of these materials in agriculture and environmental engineering to increase soil water retention. Under such conditions, there is water absorption of the SAP in soil under load. This paper presents the results of absorbency under load (AUL) of a cross-linked copolymer of acrylamide and potassium acrylate mixed at ratios of 0.3%, 0.5% and 1.0% with coarse sand and sandy loam. The mixtures were subjected to loads equivalent to 10, 20 and 40 cm of soil. The highest differences in AUL values for both soils, compared to the control sample, were obtained after 24 hours and at a maximum load of 5.9 kPa, which corresponds to a load of a 40 cm thick topsoil layer. The AUL was 71.4 g∙g^−1^ for coarse sand and 52.7 g∙g^−1^ for sandy loam with a 1.0% SAP addition, which corresponded to 24.0% and 18.0%, respectively, of the absorption in the control sample. All the conducted tests revealed a significantly low rate of water absorbency, which is especially important for capturing the water that infiltrates into the soil profile. The results demonstrate that water absorption by SAPs decreased with the increase in SAP addition.

## Introduction

SAPs are crosslinked polymer networks with strong hydrophilic groups that can be tailored to meet specific parameters of water absorption, depending on the composition^1,2^. The loosely cross-linked structure of SAPs and their capability to absorb water and its solutions in amounts several hundred times higher than their dry mass have attracted much attention in recent years^3–6^. Because the absorption capacity of SAPs exceeds that of conventional products, SAPs are widely used in medicine, the manufacturing of personal care products, water treatment, food storage, environmental engineering, forestry, and a variety of agricultural and urban greenery applications as soil conditioning agents^7–14^. The function of SAPs in the soil is to absorb water from rainfall or irrigation systems and then gradually release it to plants to support their growth and enhance their quality^15^. Chemical crosslinks prevent the dissolution of SAPs in water during the swelling process^16,17^. Because they do not become hydrophobic during droughts, these materials may support water retention and improve the water retention capacity (WRC) of sandy soils and permeable soils much more efficiently than can humic substances^18^. As a result, SAPs are characterized by properties that enable them to support vegetation growth and reduce evaporation and leaching of fertilizers and protective agents in areas with limited irrigation opportunities, where irrigation is necessary to support plant development and reduce water stress^19–21^. The water absorbed by SAPs may be supplied to plants by suction forces of the roots, so even up to 95% of the water can be used^22^. Water absorption by SAPs in soil depends on several factors, including soil structure, saline content, temperature, pH, moisture content, and presence of microorganisms, as well as soil wetting and drying cycles^23^. On the other hand, in terms of surface performance characteristics, the amendment type does have a significant effect on certain soil parameters, e.g., shear strength, soil structure and total soil porosity^24–28^.

Considering the target application of SAPs, the swelling kinetics of the absorption process is very important. When the SAPs are submerged in water, the water diffuses into the polymer matrix, and the material starts swelling. The water continues to migrate to the dynamically emerging spaces between macromolecular chains until the equilibrium state is achieved^29^. The water transport in SAPs is Fickian in nature^30^. As far as SAPs are concerned, the spring and dashpot Voight-based viscoelastic model is best known and most often applied for use in modeling creep and relaxation^31–33^.

The most commonly used way of introducing SAPs into soil is mechanical mixing with soil at the required depth^34^. Regardless of the manner of introduction to the soil, the SAP will swell under loading and in a limited space. The topsoil layer load directly influences the reduced absorption capacity^35,36^.

However, so far, few studies have been conducted that describe the characteristics of the interactions between SAPs and soil. The external load and the swelling capability of SAPs in porous regions of the soil have a direct influence on the SAP’s water absorption capacity. The described results of absorbency under load (AUL) either include the basic parameters of synthesized SAPs or refer to their applications in personal care products^31–33,37^. The results of these studies do not reflect the characteristics of SAP swelling in soil and thus cannot be representative of applications in environmental engineering and agriculture.

The main objective of the present research project is to provide an analysis of the SAP liquid absorption in soil mixtures under loads corresponding to various topsoil layer thicknesses. The authors prepared mixtures of SAPs and soils of various densities and at various ratios. The mixtures were subjected to additional loads with solid weights that simulated upper soil layers of various thicknesses. The objective of the study was to describe the process of SAP swelling in time in conditions similar to the actual applications in environmental engineering and agriculture.

## Materials and Methods

### Materials

A cross-linked copolymer of acrylamide and potassium acrylate – Aquasorb 3005 KL (SNF FLOERGER, Andrézieux, France) was used in this study. The SAP used in the experiments was in the form of dry, irregularly shaped granules. The SAP-soil mixture was prepared using two types of soil: coarse sand and sandy loam. The samples were collected from the upper soil horizon (0–30 cm) at the Experimental Field Station of the Wrocław University of Environmental and Life Sciences in Swojec (51°06′58″N 17°08′26″E).

### SAPs and physical characteristics of the soils

The grain size distribution of the SAPs and soils used was determined by the sieving method. A total of 500 g of dry material was sieved by pouring onto a sequence of 6 sieves with mesh widths of 0.10, 0.25, 0.50, 1.00, 2.00, and 5.00 mm. Soils that contained finer particles were subjected to hydrometer analysis. The tests were repeated 3 times for each material, and the results were averaged. Soil bulk densities were determined by the bulk density test, in which the weight and volume of the soil sample were measured after being dried in the oven. Soil organic matter (SOM) was determined as percentage weight loss before and after burning in a 400 °C furnace. As a result, grain size distribution curves with a range of grains were created. The soil parameters are presented in Table 1. The tested soils were classified according to the USDA classification.

**Table 1:** Physical characteristics of soils and SAP.

Soil type	BD [g∙cm^−3^]	SOM [%]	Grain size distribution [%]		
			Clay [<0.002 mm]	Silt [0.002–0.05 mm]	Sand [0.05–2.00 mm]
Sandy loam	1.65	2.80	9.00	14.00	77.00
Coarse sand	1.80	1.00	0.00	0.00	100.00

BD - soil bulk density; SOM - soil organic matter.

### AUL measurement procedure

The tests were based on the measurement concept developed by Ramazani- Harandi *et al*.^38^ and were modified by Lejcuś *et al*.^35^. Tests of AUL were conducted in a Multitest 2.5-xt apparatus manufactured by Mecmesin. A mixture of dry soil (200 g) with SAP in predefined proportions (0.30%, 0.50%, 1.00%) was prepared separately for each test. The SAP was manually mixed with the soil in a container using a mixer until a homogeneous mixture was obtained. A set of Plexiglas cylinders was prepared for the tests. The SAP-soil mixture was placed in the internal cylinder with a porous bottom (d = 70.00 mm) and subjected to the cylindrical solid load (d = 68.00 mm) of a weight corresponding to the upper soil layer thickness. The whole set was then placed in the external cylinder (d = 80.00 mm) on the tray of the apparatus. Afterwards, distilled water at a temperature of 23 °C was added to the external cylinder through a droplet system. The water level in the external cylinder was regulated to reach the height of the sample to ensure full saturation for the duration of the experiment. The amount of water during the tests was also controlled to prevent the hydraulic pressure from influencing the sensor device. In the experiment, the SAP-soil mixture was placed under a cylindrical solid load with a weight corresponding to layers of soil with a bulk density of 1.50 g/cm^3^ and heights of 10.00, 20.00, and 40.00 cm. The weights were converted into kPa. These heights correspond to the typical layers of covers of earth structures or to those layers used when planting trees and bushes. The experiment was repeated three times for each SAP-soil mixture-load configuration. The results were averaged.

The load caused by the soil layer was calculated using the following equation:

1 $${\rm{\upsilon }}={\rm{\rho }}\cdot {\rm{g}}\cdot {\rm{h}}[P{\rm{a}}]$$

where υ is the soil load [Pa], ρ is the bulk density of the overlying soil [kg∙ m^−3^], g is the acceleration due to gravity [m∙s^−2^], and h is the soil layer height [m].

**Figure 1:**
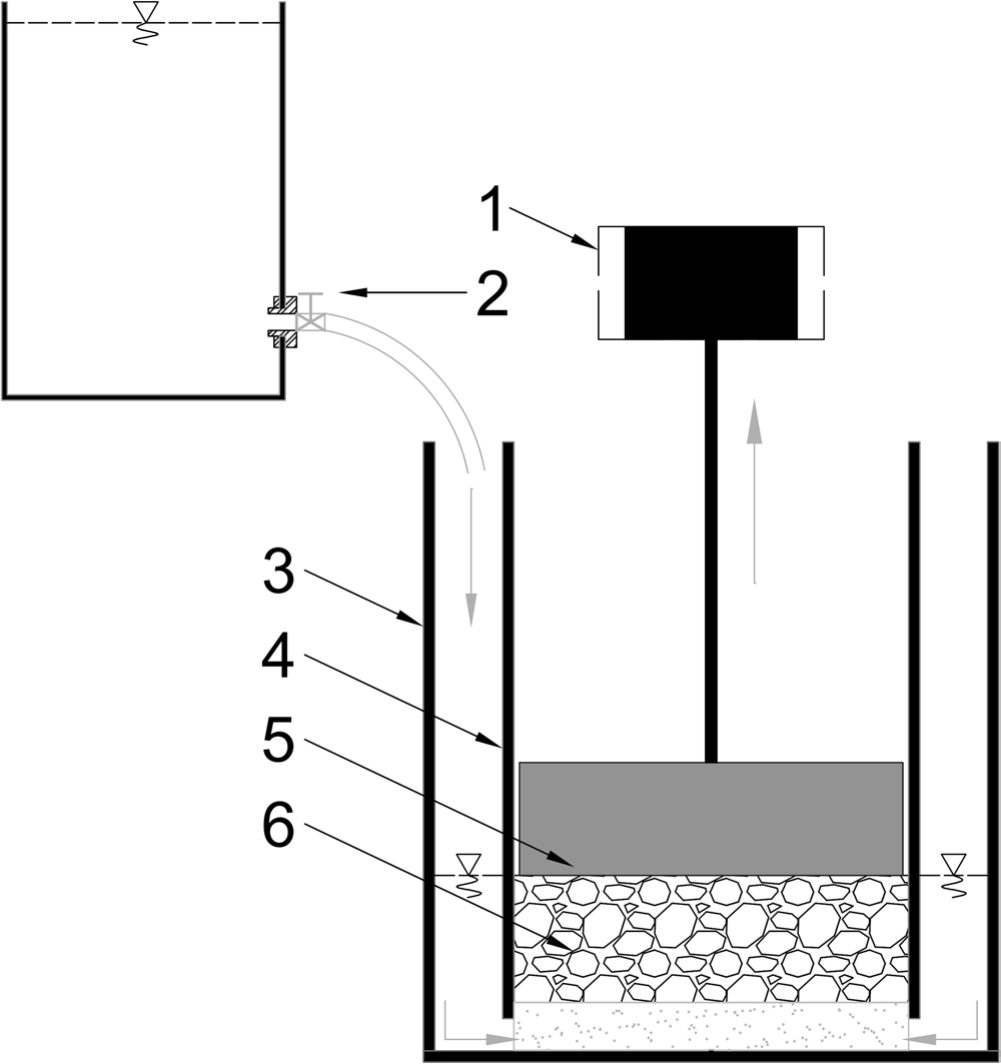
Test apparatus (Mecmesin multitest-2.5xt) with the experimental setup. (1) ICL Force sensor, (2) droplet system, (3) outer cylinder with water, (4) inner cylinder with porous bottom, (5) load, (6) SAP-soil mixture.

The changes in sample height were measured by an apparatus sensor for 24 hours and plotted as displacement over time. To simulate the soil load, the cylindrical metal weights were placed on top of the SAP-soil mixture so as to prevent rubbing against the walls of the cylinder. Measurements were taken with the use of a force sensor (ICL) with an accuracy of ±0.10%. During swelling under load, the sample put pressure on the ICL force sensor. When the force measured by the sensor reached 0.20N, the sensor moved up until the force was reduced to 0.00N. The displacement of the sensor and increase in force were measured at a frequency of 10 Hz by the controlling software Emperor™ (version 1.18-305, Mecmesin Ltd., Slinfold, United Kingdom). This procedure allowed us to measure the increase in sample height and the time of swelling of the SAP. Additional tests were conducted using pure SAP without load as a control sample. The drawing of the apparatus with the experimental setup is presented in Fig. 1.

The measured increase in sample height was converted into swelling value [g∙g^−1^]. The test series were conducted in the same way. AUL was calculated according to the following formula (2)^38^:

2 $${\rm{AUL}}=\frac{{\rm{W}}2-{\rm{W}}1}{{\rm{W}}1}$$

where AUL [g∙g^−1^] is absorbency under load, and W_1_ [g] and W_2_ [g] are the weights of the swollen SAP and dry SAP, respectively.

The data were processed using the Voight-based model, and the swelling rate of the SAP was described by the following Eq. (3)^32,33^:

3 $${{\rm{S}}}_{{\rm{t}}}={{\rm{S}}}_{{\rm{e}}}(1-{e}^{-\frac{t}{\tau }})$$

where S_t_ [g∙g^−1^] is the swelling at time t, S_e_ [g∙g^−1^] is the equilibrium swelling (power parameter, absorption capacity), t [min] is the time of swelling, and τ [min] is the time parameter (time required to reach 63% of the maximum absorption capacity).

Using the Voight-based model, the rate parameter (τ) and equilibrium swelling (S_e_) were calculated for each load-ratio configuration.

Statistical analyses were conducted to determine the statistical significance of the obtained swelling results of SAP-soil mixtures under load. The equality of variances was calculated using the Levene test.

The statistical groups (different loads and SAP-soil proportions) for both soils used were compared with the control group (without load or soil) by performing Dunnett’s C test (post hoc group comparison). The level of significance was set at 0.05 for each group (n = 9). Data analysis and curve fitting were conducted using Statistica software (version 13.0, StatSoft, Kraków, Poland) and Microsoft Excel (version 2007, Microsoft Corporation, Redmont, USA).

## Results

### Physical characteristics of soils and SAPs

**Figure 2:**
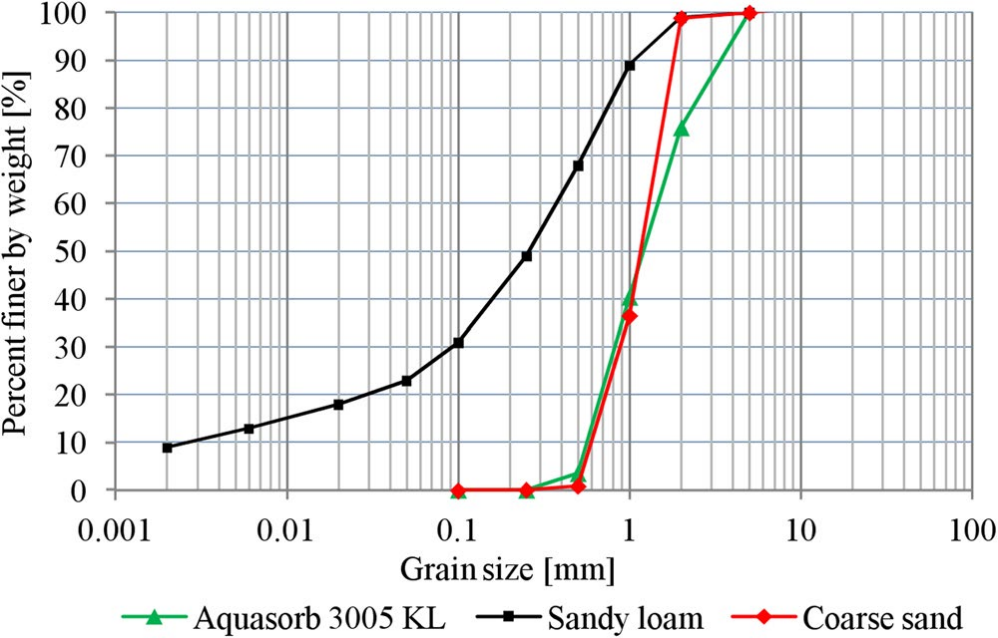
Grain size distributions of the tested SAP and soil.

The characteristics of grain size distribution need to be evaluated to determine the correct methods for swelling capacity characterization. The most important property of SAPs, i.e., their absorption capacity, is directly linked to particle size and degree of cross-linking^39^. The grain size distribution of soils is also important from the point of view of preparing SAP-soil mixtures. The grain size distributions of the analyzed soils and SAP is presented in Fig. 2. For Aquasorb 3005 KL, particles from the 0.50–1.00 mm range account for the highest share (over 36%), followed by the 1.00–2.00 mm particles (35.00%). For coarse sand, the highest share was noted for the 1.00–2.00 mm particles (over 62.00%), while particles in the 0.50–1.00 mm range accounted for more than 35%. Sandy loam was characterized by the most differentiated particle sizes. The ranges of 0.50–1.00 mm, 0.25–050 mm and 0.10–0.25 accounted for 21.00%, 19.00% and 18.00%, respectively.

### AUL and swelling kinetics

**Figure 3:**
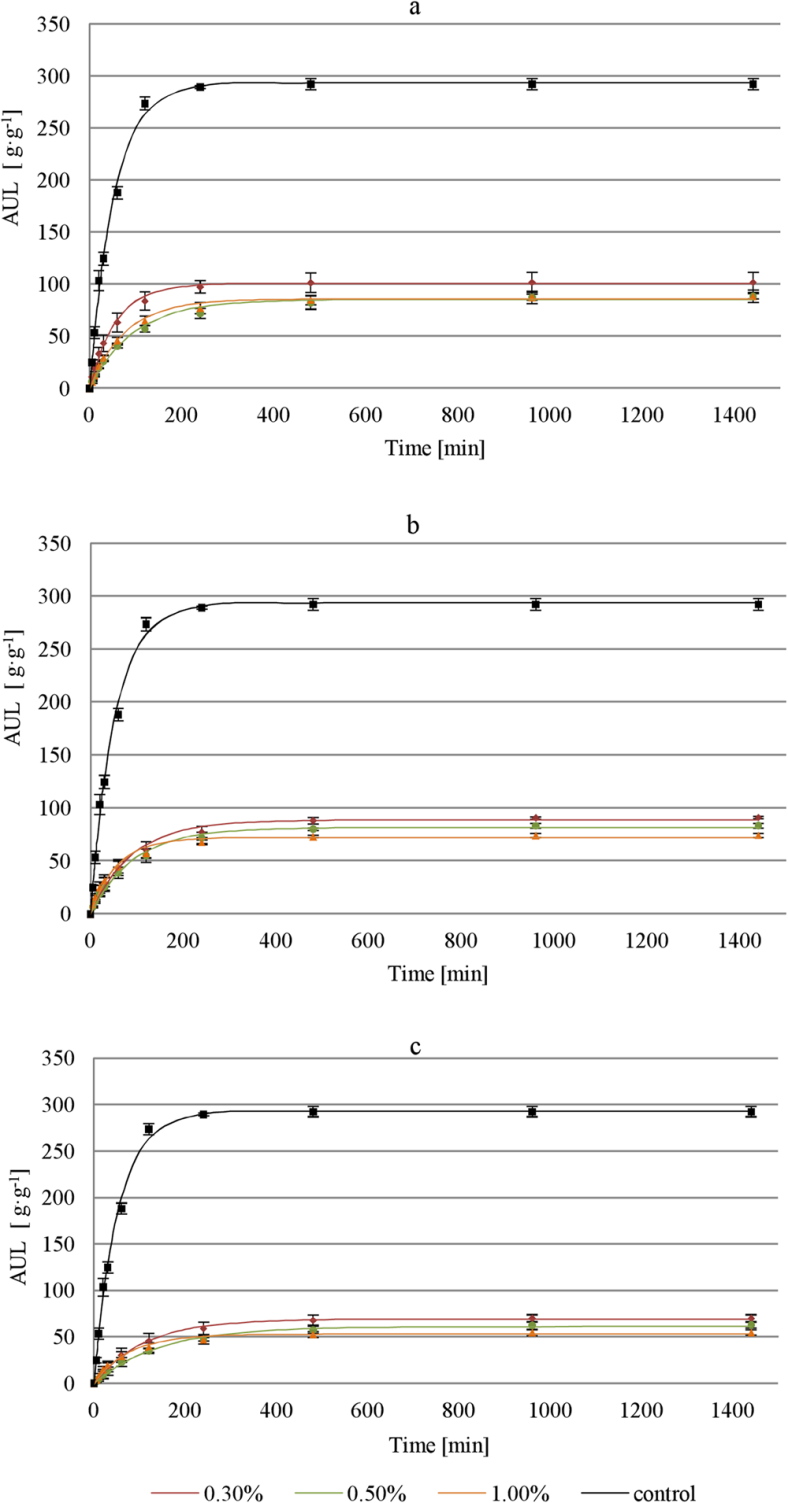
Time-dependent absorption under load for SAP and sandy loam mixtures, under (**a**) 10.00, (**b**) 20.00, and (**c**) 40.00 cm thick soil layer with bulk density of 1.5 g∙cm^−3^ (error bars: standard deviations).

**Figure 4:**
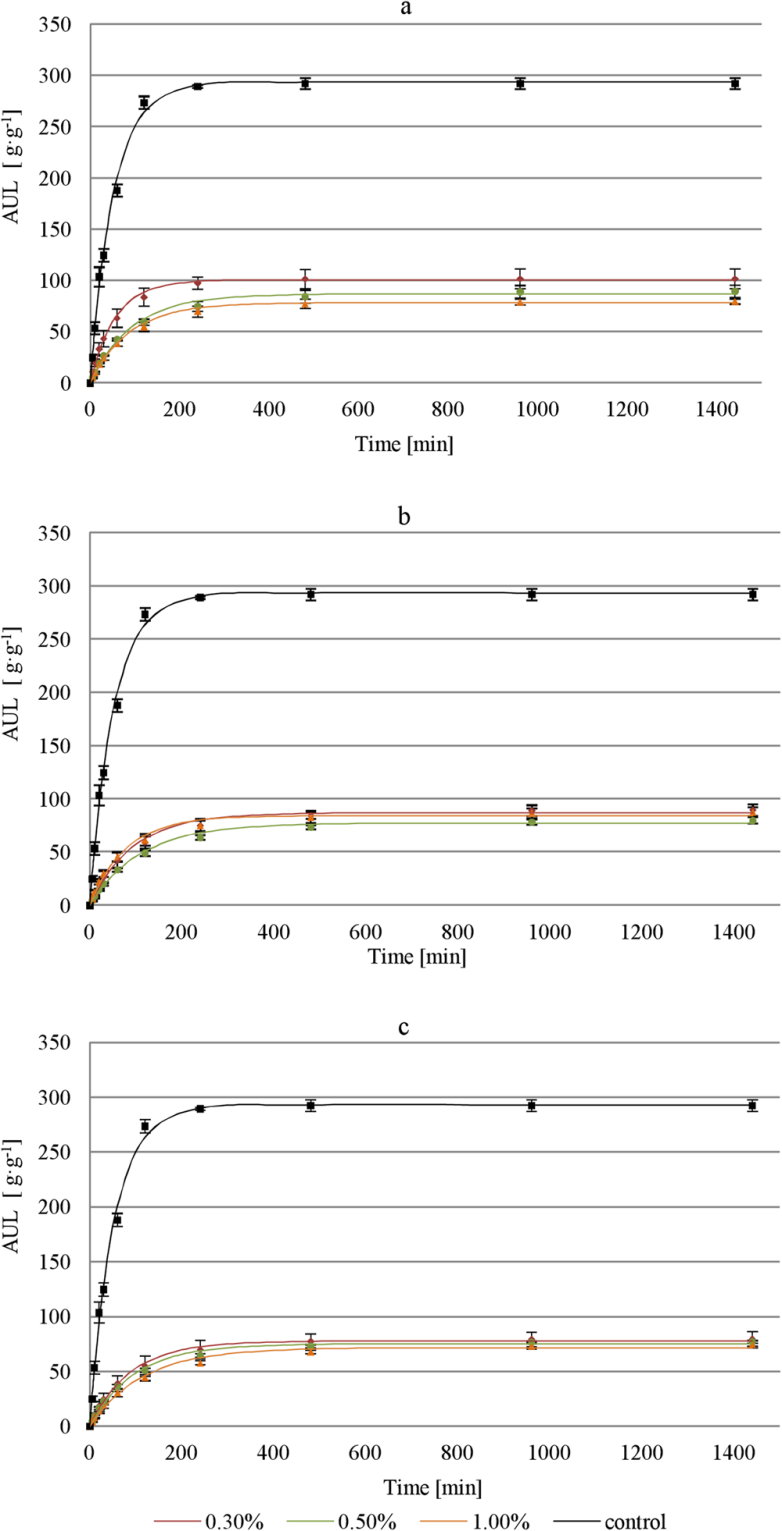
Time-dependent absorption under load for SAP and coarse sand mixtures under (**a**) 10.00, (**b**) 20.00, and (**c**) 40.00 cm thick soil layer with a bulk density of 1.50 g∙cm^−3^ (error bars: standard deviations).

The obtained values of the rate parameter (τ) and equilibrium swelling (S_e_) for the tested SAP-soil mixtures under various loads are presented in Table 2, and the curves fitted to the swelling process are shown in Figs. 3 and 4.

**Table 2:** Rate parameter (τ) and equilibrium swelling (S_e_) for the tested SAP.

Soil	SAP-Soil proportion [%]	Topsoil layer height [cm]	Load [kPa]	τ [min]	Se [g/g]	Standard error of regression [g/g]	Coefficient of determination R^2^ [−]
Sandy loam	0.30	10.0	1.47	95.91	96.52	2.53	0.996
		20.0	2.94	90.18	88.59	3.77	0.990
		40.0	5.89	110.16	69.14	1.04	0.999
	0.50	10.0	1.47	83.58	81.61	1.85	0.997
		20.0	2.94	93.61	81.53	2.87	0.993
		40.0	5.89	142.76	61.48	1.49	0.997
	1.00	10.0	1.47	76.36	76.22	2.54	0.994
		20.0	2.94	57.25	71.88	3.42	0.987
		40.0	5.89	75.97	52.68	2.21	0.990
Coarse sand	0.30	10.0	1.47	55.99	100.28	2.75	0.996
		20.0	2.94	94.09	86.58	2.92	0.994
		40.0	5.89	88.57	77.94	2.08	0.996
	0.50	10.0	1.47	96.51	84.80	3.50	0.991
		20.0	2.94	111.91	76.88	2.40	0.995
		40.0	5.89	97.12	75.40	2.81	0.992
	1.00	10.0	1.47	77.89	85.53	2.37	0.996
		20.0	2.94	79.09	84.13	3.26	0.992
		40.0	5.89	115.81	71.43	2.97	0.990
Control without load		0.00	0.00	52.37	293.19	6.47	0.997

The analysis of results demonstrated that the assumptions for parametric tests (variance equality and normal distribution) were not fulfilled. The non-parametric Dunnett’s C test was conducted to compare individual groups with the control group. The results revealed that all variants of the experiment (different loads and SAP-soil proportions) differed substantially from the control samples.

The results prove that load has a noticeable influence on the reduction in swelling of SAP-soil mixtures. Regardless of the applied load and the proportion in which the SAP was mixed with soil, the differences in water absorption compared to those in free swelling are significant (Table 2; Figs. 3 and 4).

The highest differences in the AUL values compared to the control samples after 1440 min, for both coarse sand and sandy loam, were obtained for the pressure of 5.89 kPa, which corresponds to a load of topsoil layer thickness of 40.00 cm (bulk density of 1.50 g∙cm^−3^) (Figs. 3 and 4). In this variant, the obtained AUL values were 71.40 g∙g^−1^ for coarse sand and 52.70 g∙g ^−1^ for sandy loam (1.00% SAP), which account for over 24.00% and nearly 18.00%, respectively, in comparison to the control group. The load affects not only the AUL but also the swelling kinetics. A sample of SAP-coarse-sand mixture (1.00%) subjected to a load of 5.89 kPa reached an AUL of 16.30 g∙g^−1^ after the first 30 min of the experiment, while it took the control sample only 3 min to reach this value. For the same load and the proportion of 0.50% in the SAP-sandy-loam mixture, the AUL after 30 min was 11.70 g∙g^−1^, while the control sample achieved this value after only 2 min.

The highest AUL values were noted for samples loaded with a 10 cm soil layer (1.47 kPa), where the share of SAP in the mixture was the lowest (0.30%) (Figs. 3 and 4). The maximum AUL values in this configuration were 100.30 g∙g ^−1^ for coarse sand and 96.50 g∙g ^−1^ for sandy loam. Although the AUL results for both soils were similar, the swelling rates differed. The SAP-sandy-loam sample took 220 min to reach 90.00% of the total AUL, while the SAP-coarse-sand mixture sample needed only 130 min. Regardless of the type of soil, the proportions of SAP in the mixture and of the external load, the amount of retained water and the absorption rate were significantly lower than those in the control group. The difference in absorption under load between the SAP-sandy-loam mixture (0.50%) subjected to a load of 5.89 kPa and the control sample without load exceeds 1200.00%. The lowest variability of the differences in absorption was noted for the smallest load of the coarse sand sample (0.30%), which equaled approx. 300.00% for the entire duration of the experiment. To obtain the maximum efficiency of water absorption by the SAP-soil mixture, one should take into account the share of SAP in the mixture and the thickness of the layer with which it will be loaded.

## Discussion

The tests of SAP in terms of their capacity to absorb water and its solutions are based on various methodological approaches and usually consist of two main types: water absorbency tests, where the SAP is directly mixed with soil, and tests of absorbency under load, where the SAP is subjected to additional load. The present study combines both these approaches and focuses on analyzing the absorbency under load for SAP-soil mixtures at various proportions to obtain the actual values of water absorption by the given SAP under a specific load. The comparison of experiments conducted on SAP-soil mixtures in various proportions reveals that the water absorption by the SAPs decreased with the increase in SAP addition^20,40–42^. In cases where this correlation is not true, the differences in water absorbency are low and reach only approx. 5.00%^43^. On the other hand, experiments conducted on SAP samples subjected to direct load confirm the influence of load on the absorption value. However, in this approach, the type of absorbed substance is an important factor. The results of the present experiment and of others conducted with use of DI water demonstrate that the AUL decreases significantly with the increase in load^35^, while tests on the absorption of saline solutions yielded less varied results, although the differences in the load of the sample were three times higher^36,44^. Although numerous studies on various SAP polymers have been conducted, the comparison of results still poses a problem. Due to the differences in the methodologies used by the authors, the conditions of the experiments and the use of various types of SAPs in various doses, the differences in the results are often significant and prevent a direct comparison of the obtained results. Table 3 presents the results obtained by other authors that take into account the SAP addition in SAP-soil mixtures. Table 4 presents the results obtained by other authors that take into account the absorbency under load. The values of load, SAP addition, and water absorbency have been converted to the same units.

**Table 3:** Comparison of water absorbency results for practical applications of SAPs with various soil additions.

Authors	Type of SAP	Type of soil	Soil bulk density [g∙cm^−3^]	Absorbed substance	Load [kPa]	SAP addition [% w/w]	Water absorbency [g∙g^−1^]
Akhter *et al*.^43^	Polymerization of acrylamide (N,N-methyl-bis-acrylamide) mixed Na and K salts of acrylic acid	Sandy loam	N/a	DI water	—	0.1	34.00
						0.2	36.00
						0.3	42.00
Agaba *et al*.^40^	Liquasorb	Sandy loam	1.41	Water	—	0.2	181
						0.4	89.08
Leciejewski^41^	Super Absorbent Plus	Loamy sand	1.41	DI water	—	0.2	148.05
			1.29			0.3	103.2
Abedi-Kupai *et al*.^20^	PR3005A	Sandy loam	1.78	Tap water (EC = 0.2 dS∙m^−1^)	—	0.2	217.6
						0.4	126.45
						0.6	95.24
						0.8	85.46
Essawy *et al*.^46^	Crosslinked (CTS/Cell)-g-PAA	Sand	N/a	DI water	—	2.0	390
Yu *et al*.^42^	GNKH (60% Acrylic, 40% Acrylamide)	Loamy clay	N/a	Tap water (EC = 0.5 dS∙m^−1^)	—	0.1	375.70
						0.5	42.68
						1.0	23.59
						2.0	17.81

**Table 4:** Comparison of water absorbency results for practical applications of SAPs under various loads.

Authors	Type of SAP	Type of soil	Soil bulk density [g∙cm^−3^]	Absorbed substance	Load [kPa]	SAP addition [% w/w]	Water absorbency [g∙g^−1^]
Lawal *et al*.^47^	Xerogel	—	—	DI water	4.5	—	27.00
Salimi *et al*.^44^	Carrageenan-based SAP hydrogel hybrid (AA/HEA = 1)	—	—	0.9% NaCl solution	2.07	—	38
					6.21		34
Pourjavadi *et al*.^36^	CMC-g-poly (acrylic acid-co-2-acrylamido-2-methylpropanesulfonic acid)/silica gel composite	—	—	0.9% NaCl solution	2.07	—	44
					4.14		40
					6.21		40
Lejcuś *et al*.^35^	Aquasorb 3005 KL	—	1.3	DI water	1.28	—	63.4
					2.55		21.9
					3.83		19.3

In spite of the differences mentioned before, the results obtained by the authors of this study are similar to those obtained by other authors. The addition of SAPs to soils improves the retention of water, which may be used by plants. However, the water absorbency values decrease with the increase in the SAP-soil ratio, which should be considered when choosing the dosage of SAP. Another important parameter is the influence of the external load, which directly limits the absorption volume. The conducted research confirmed that tests of SAP-soil mixtures result in higher AUL values than tests of SAP without subjecting the soil to load. This difference results from the swelling of single grains of SAP in soil pores. In this way, the conducted research reflected the interaction between SAP and soil with which the material is mixed and additionally loaded with an external layer. The obtained results also illustrate the necessity to consider the influence of soil load on the SAP and the resulting water absorbency reduction and rate if SAPs are used as a means to improve soil water retention. This problem may be solved by enabling the SAPs to absorb water in the soil medium without the influence of the load of a layer of soil, e.g., in the form of water-absorbing geocomposites, whose structure provides the SAPs with sufficient space to swell freely^45^.

## Conclusions

The conducted research confirmed that both AUL and SAP content are important parameters that determine water absorbency by SAPs in soil medium, which in turn is reflected in the efficiency of their use in environmental engineering and agriculture. The main factors that affect water absorbency by SAPs are the depth of application and soil bulk density, which determine the influence of load on the SAP and the amount of its addition to soil. The obtained results show that the effectiveness of SAP application into soil may be estimated based on the results of water absorbency of SAP-soil mixtures. In the conducted tests, the differences in the amount of water retained (absorbed) by the SAP mixed with soil, for the analyzed maximum load 5.89 kPa, exceed 1200% in comparison to the control sample. Soil load not only limits water absorption by the SAP, but it also delays swelling time, which is important if the superabsorbent is applied as an additive to improve soil water retention. The obtained results confirm the need to reconsider the manner of introducing SAPs into the soil. These results also indicate the need to specify the depth of application and the manner of introducing SAPs to soil in the descriptions of field and laboratory tests.
